# A Novel Small RNA, DsrO, in Deinococcus radiodurans Promotes Methionine Sulfoxide Reductase (*msrA*) Expression for Oxidative Stress Adaptation

**DOI:** 10.1128/aem.00038-22

**Published:** 2022-05-16

**Authors:** Yun Chen, Mingming Zhao, Mengli Lv, Min Lin, Jin Wang, Kaijing Zuo

**Affiliations:** a Single Cell Research Center, School of Agriculture and Life Sciences, Shanghai Jiao Tong University, Shanghai, China; b Biotechnology Research Institute, Chinese Academy of Agricultural Sciences, Beijing, China; University of Illinois at Urbana-Champaign

**Keywords:** *Deinococcus radiodurans*, oxidative stress, MsrA, DsrO, small RNA, posttranscriptional regulation

## Abstract

Reactive oxygen species (ROS) can cause destructive damage to biological macromolecules and protein dysfunction in bacteria. Methionine sulfoxide reductase (Msr) with redox-active Cys and/or seleno-cysteine (Sec) residues can restore physiological functions of the proteome, which is essential for oxidative stress tolerance of the extremophile Deinococcus radiodurans. However, the underlying mechanism regulating MsrA enzyme activity in D. radiodurans under oxidative stress has remained elusive. Here, we identified the function of MsrA in response to oxidative stress. *msrA* expression in D. radiodurans was significantly upregulated under oxidative stress. The *msrA* mutant showed a deficiency in antioxidative capacity and an increased level of dabsyl-Met-S-SO, indicating increased sensitivity to oxidative stress. Moreover, *msrA* mRNA was posttranscriptionally regulated by a small RNA, DsrO. Analysis of the molecular interaction between DsrO and *msrA* mRNA demonstrated that DsrO increased the half-life of *msrA* mRNA and then upregulated MsrA enzyme activity under oxidative stress compared to the wild type. *msrA* expression was also transcriptionally regulated by the DNA-repairing regulator DrRRA, providing a connection for further analysis of protein restoration during DNA repair. Overall, our results provide direct evidence that DsrO and DrRRA regulate *msrA* expression at two levels to stabilize *msrA* mRNA and increase MsrA protein levels, revealing the protective roles of DsrO signaling in D. radiodurans against oxidative stress.

**IMPORTANCE** The repair of oxidized proteins is an indispensable function allowing the extremophile D. radiodurans to grow in adverse environments. Msr proteins and various oxidoreductases can reduce oxidized Cys and Met amino acid residues of damaged proteins to recover protein function. Consequently, it is important to investigate the molecular mechanism maintaining the high reducing activity of MsrA protein in D. radiodurans during stresses. Here, we showed the protective roles of an sRNA, DsrO, in D. radiodurans against oxidative stress. DsrO interacts with *msrA* mRNA to improve *msrA* mRNA stability, and this increases the amount of MsrA protein. In addition, we also showed that DrRRA transcriptionally regulated *msrA* gene expression. Due to the importance of DrRRA in regulating DNA repair, this study provides a clue for further analysis of MsrA activity during DNA repair. This study indicates that protecting proteins from oxidation is an effective strategy for extremophiles to adapt to stress conditions.

## INTRODUCTION

Reactive oxygen species (ROS) accumulation is the indirect consequence of failed or inefficient electron-transfer processes (1). Nearly all organisms produce ROS when they are exposed to UV light, gamma and X-rays, and abiotic and biotic stresses (2). ROS such as H_2_O_2_ can produce hydroxyl radicals that oxidize both bases (adenine and guanine) and ribose moieties of DNA. Damaged guanine allows its electrons to enter into nearby oxidized bases, producing a wide variety of DNA lesions (3, 4). The polyunsaturated fatty acids of cell membranes are easily peroxidated in some bacteria, producing the cytotoxic compound 4-hydroxy-2-nonenal (5). In addition, ROS have high oxidation reactivity toward sulfur-containing amino acids and metal-containing cofactor sites in proteins, causing irreversible inactivation of many different proteins (6). The sulfur atom in the thioether side chain of Met can be oxidized to sulfoxide, converting Met into methionine sulfoxide (Met-SO). Met-SO residues can be irreversibly further oxidized to methionine sulfone (Met-O) (7). Overall, oxidative damage caused by ROS has devastating effects on DNA, lipid structure, and protein activity in most organisms.

Deinococcus radiodurans is an extremophile with an extremely high tolerance to gamma radiation, UV irradiation, desiccation, and other oxidative stresses (8). To cope with these stressors, D. radiodurans has evolved efficient antioxidative ROS scavenging strategies. D. radiodurans has a relatively low number of respiratory chain enzymes and induces the glyoxylate bypass of the tricarboxylic acid cycle to reduce endogenous ROS production (9). The antioxidant compound deinoxanthin provides D. radiodurans with a distinctive pale brown color and endows it with high ROS-elimination capacity together with manganese (10). With the help of the DNA recombination repair system (RecA, PprA, and other recombination regulators), D. radiodurans displays a remarkable ability to reverse DNA damage and reconstitute the genome (11). D. radiodurans is also well equipped with additional ROS scavenging enzymes (e.g., catalase and superoxide dismutase) (12). These different strategies work effectively to restore damaged D. radiodurans cells to a normal physiological state. Although the molecular mechanism of DNA repair has been described in detail (13–15), the details of the regulatory mechanism involved in recovering ROS-oxidized protein function in D. radiodurans are unclear.

By comparing key enzymes related to amino acid biosynthesis among different bacterial species using Kyoto Encyclopedia of Genes and Genomes (KEGG) pathway analysis, enzymes in the methionine biosynthesis pathway were found to be incomplete in D. radiodurans (16). Culture experiments in media without methionine confirmed that D. radiodurans CGMC1.3828T requires exogenous methionine for growth (16). Accumulating evidence indicates that the level of protein oxidation is negatively correlated with the survival of irradiated bacteria (17, 18). Protein oxidation is likely an important cause rather than a consequence of radiation toxicity; thus, the ability to protect proteins against oxidation to a large extent distinguishes radiation-resistant from radiation-sensitive *Deinococcus* species (18–20). Because Met residues are particularly sensitive to oxidative damage, the limited Met resources in D. radiodurans likely contribute to a stronger ability to rescue oxidized proteins compared with sensitive bacteria (21).

Methionine sulfoxide reductase (Msr) is a key enzyme in reducing oxidized Met residues in proteins to restore their function (22). Bacterial mutants for *msrA* and/or *msrB* display ROS-sensitive phenotypes. The two forms of Msr that reduce l-Met-S-(O) are MsrA and MsrB; MsrA functions on both peptide-bound l-Met-S-(O) and free l-Met-S-(O) (23, 24). Msr proteins are evolutionarily conserved and function similarly in response to ROS exposure. Msr expression is controlled by alternative sigma transcription factors in different bacterial species (25, 26). D. radiodurans contains two types of Msr proteins, MsrA and MsrB. MsrB is less efficient due to its higher km-MetSO values and slower turnover in Trx/TrxR-based assays (27, 28). It is therefore likely that MsrA proteins in D. radiodurans are the primary protein reducer under oxidative stress, although the regulatory mechanism of *msrA* is unknown. In this study, we found that a small RNA (sRNA), DsrO, probably acting in concert with the transcription factor DrRRA, regulates *msrA* expression for protein translation in D. radiodurans exposed to oxidative stress.

## RESULTS

### *msrA* expression in D. radiodurans is strongly induced by oxidative stress.

**FIG 1:**
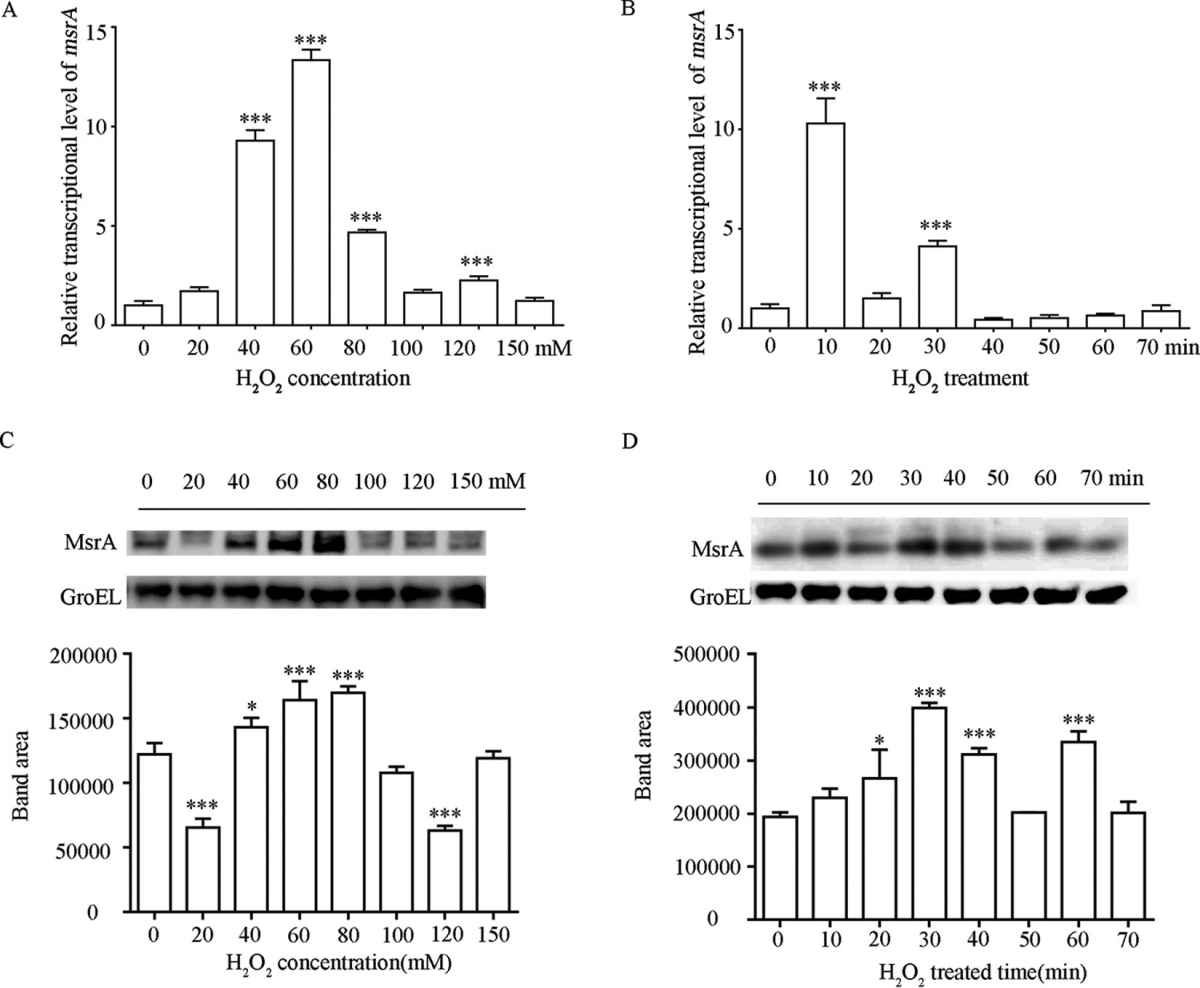
*msrA* expression is strongly induced by oxidative stress in D. radiodurans. (A and C) *msrA* transcriptional level (A) and protein level (C) in D. radiodurans treated with different concentrations of H_2_O_2_ for 30 min. (B and D) *msrA* transcriptional level (B) and protein level (D) in D. radiodurans treated with 80 mM H_2_O_2_ for different times. The *msrA* transcriptional level was analyzed by qRT-PCR with 16S rRNA as the reference gene. GroEL was used as the internal control for Western blotting. Asterisks indicate statistically significant differences of the value compared to that of untreated cells (one-way analysis of variance [ANOVA], Dunnett’s multiple-comparison test; ***, *P* ≤ 0.001; *, *P* ≤ 0.05). The experiments were performed at least three times, and the data are presented as means ± standard error of the mean (SEM).

Methionine sulfoxide reductases (Msr proteins) repair oxidized methionine residues of damaged proteins and are found in nearly all organisms (29). Bacterial mutants lacking Msr proteins are often sensitive to oxidative stress; in pathogenic bacteria, the mutants exhibit decreased virulence via reduced survival probability in host cells (29). To analyze the function of MsrA in D. radiodurans during oxidative stress, we cultured D. radiodurans R1 cells in growth medium with different concentrations of hydrogen peroxide (H_2_O_2_) (Fig. 1). The real-time quantitative PCR (qRT-PCR) results showed that *msrA* mRNA expression was significantly upregulated by H_2_O_2_ exposure, with the highest expression (13-fold higher than that of the untreated control) in medium containing 60 mM H_2_O_2_ (Fig. 1A). However, when H_2_O_2_ concentrations were higher than 80 mM, *msrA* transcript levels were unchanged compared to untreated cells. Further analysis of *msrA* temporal expression in D. radiodurans R1 treated with 80 mM H_2_O_2_ indicated that the expression of *msrA* reached the maximum level after 10 min (Fig. 1B). *msrA* expression was also induced by exogenous treatments with UV light (for 11 min), heat (48°C for 5 h), or cold (4°C for 5 h) stress, suggesting that *msrA* responds to a variety of different stresses (see Fig. S1 in the supplemental material).

Maintenance of increased MsrA protein synthesis levels over time is critical for the functions of antioxidative enzyme activity. We thus investigated whether MsrA protein levels in D. radiodurans R1 correspond to changes in *msrA* gene expression levels under H_2_O_2_ treatment. Western blot results indicated that the highest level of MsrA occurred during exposure to 80 mM H_2_O_2_. Consistent with the gene expression results, there were no significant changes compared to the control group when the H_2_O_2_ concentration was higher than 80 mM (Fig. 1C). MsrA protein synthesis in D. radiodurans treated with 80 mM H_2_O_2_ continuously increased between 0 and 30 min compared with the control group and then obviously declined (Fig. 1D). These results indicate that the levels of both *msrA* mRNA and the MsrA protein can be quickly upregulated in response to oxidative treatments. In bacteria, mRNA levels are often tightly correlated with the levels of the corresponding protein translation over time (30). Given that the highest level of MsrA protein occurred after the peak of *msrA* mRNA levels, we hypothesized that there is a molecular mechanism of maintaining *msrA* mRNA levels in D. radiodurans to increase protein translation under oxidative stress.

### Deletion of the *msrA* gene reduces the total antioxidant capacity and tolerance to H_2_O_2_ of D. radiodurans.

**FIG 2:**
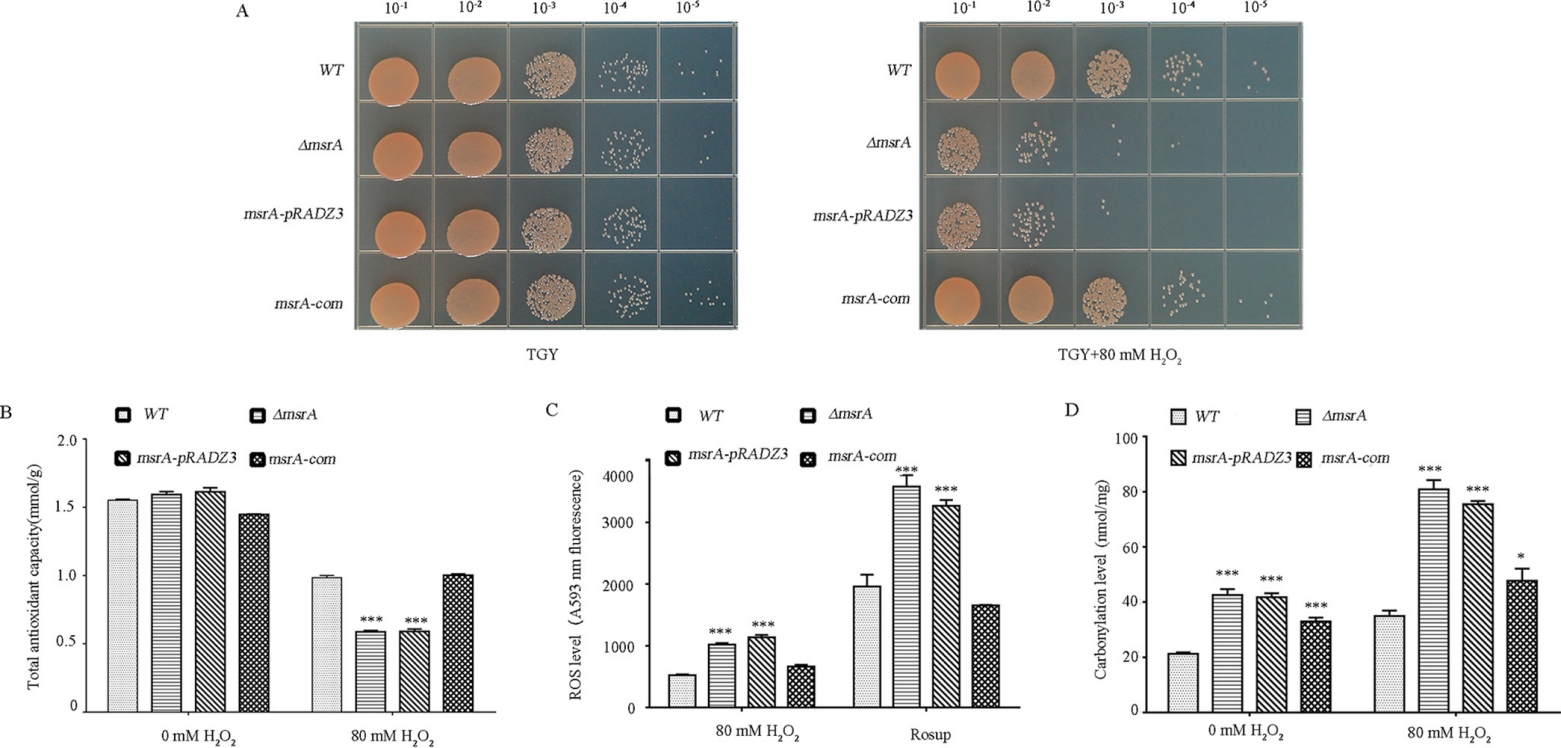
The deletion of the *msrA* gene in D. radiodurans decreases its tolerance to oxidative stress. (A) H_2_O_2_ sensitivity assays of different D. radiodurans strains. Spotted agar plates after H_2_O_2_ treatment and serial dilution. (Left) 0 mM H_2_O_2_; (right) 80 mM H_2_O_2_ for 30 min; WT, wild-type strain, Δ*msrA*, *msrA*-deleted mutant; *msrA-pRADZ3*, *msrA* mutant transformed with *pRADZ3* empty plasmid; *msrA-com*, *msrA* mutant supplemented with the *msrA* gene. (B to D) Total antioxidant capacity (B), ROS level (C), and intracellular carbonylation level (D) of different strains after H_2_O_2_ treatment. The total level of various antioxidant macromolecules, antioxidant small molecules, and enzymes in a system reflects the total antioxidant capacity of the bacterial cells. Rosup is a reactive oxygen positive-control reagent. Asterisks indicate a statistically significant difference in the value compared to that of untreated cells (one-way ANOVA, Dunnett’s multiple-comparison test; ***, *P* ≤ 0.001; *, *P* ≤ 0.05). Experiments were performed at least three times, and the data are presented as the means ± SEM.

To characterize the physiological function of MsrA in D. radiodurans, we first analyzed the growth phenotypes of the wild-type strain (WT), mock strain (Δ*msrA-pRADZ3*), *msrA* knockout strain (Δ*msrA*), and complementation strain under oxidative stress (Fig. 2). Under normal growth conditions, there was no significant difference in growth between any of the tested strains. However, under oxidative stress conditions (80 mM H_2_O_2_ for 30 min), the viability of Δ*msrA* decreased significantly compared to WT, whereas the complementation strain had a similar viability to the WT (Fig. 2A). There were no significant differences in growth among WT, Δ*msrA*, and complementary strains in response to UV light, heat, or cold stress (Fig. S2), indicating that *msrA* may not function under these stress treatments.

ROS accumulation has been suggested to be the main cause of oxidative damage, leading to decreased bacterial growth. Carbonylated protein levels can serve as an indicator of an oxidative cellular status; dysfunctional carbonylated proteins are more prone to irreversible oxidation of other amino acid residues or to the formation of protein aggregates. We therefore compared the total antioxidant capacity, ROS content, and carbonylation level in the WT, *msrA-pRADZ3*, Δ*msrA*, and complementation strains under oxidative stress. As expected, deletion of the *msrA* gene significantly reduced intracellular antioxidant capacity (Fig. 2B). In the medium with 80 mM H_2_O_2,_ ROS levels rose sharply in *ΔmsrA* and *ΔmsrA-pRADZ3* compared to the WT, supporting that MsrA acts as an oxidant scavenger under oxidative stress (31) (Fig. 2C), and in the 80 mM H_2_O_2_ treatment, *msrA* mutants had twice the level of carbonylated proteins as the WT (Fig. 2D). These data further support that MsrA is necessary for normal antioxidant capacity in D. radiodurans.

### DsrO is associated with oxidative stress, and its mutation reduces the tolerance of D. radiodurans to H_2_O_2_.

**FIG 3:**
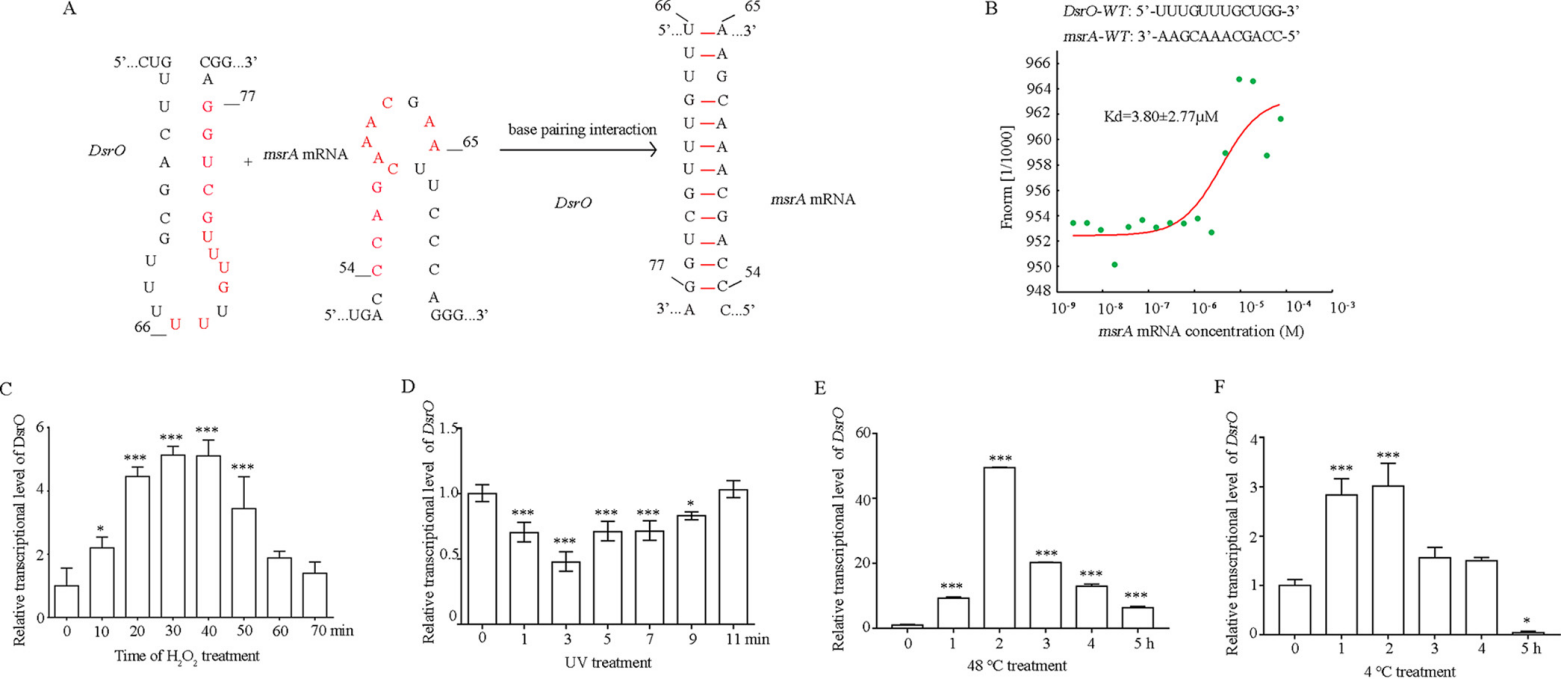
A noncoding RNA, DsrO, directly binds to the stem-loop structure of *msrA* mRNA. (A) Predicted binding pattern between *msrA* mRNA and DsrO based on the bioinformatics analysis. (B) Affinity coefficient between DsrO and *msrA* mRNA detected by the microscale thermophoresis (MST) method. (C to F) The transcriptional level of DsrO in response to the treatments of 80 mM H_2_O_2_ (C), UV radiation (D), heat (48°C) (E), and cold (4°C) (F) in D. radiodurans. Asterisks indicate statistically significant differences compared to untreated cells (one-way ANOVA, Dunnett’s multiple-comparison test; ***, *P* ≤ 0.001; *, *P* ≤ 0.05). Experiments were performed at least three times, and data are presented as means ± SEM.

Given that the peak levels of *msrA* mRNA and MsrA protein were staggered, we speculated that there is a factor such as a small RNA (sRNA) in D. radiodurans that posttranscriptionally regulates *msrA*. We thus used an sRNA target prediction program to screen sRNAs potentially binding to *msrA* mRNA using D. radiodurans genomic and transcriptomic data (RNAalifold, http://rna.tbi.univie.ac.at/cgi-bin/RNAWebSuite/RNAalifold.cgi) (32). Among the 23 sRNAs associated with the oxidative response, Dsr19 was predicted to be most likely to bind to the stem-loop region of *msrA* mRNA through a 12-nucleotide (nt) complementary fragment (5′-UUUGUUUGCUGG-3′) (Fig. 3A and B). To clearly show the characteristics of Dsr19 during oxidative stress, we renamed Dsr19 DsrO (Deinococcus radiodurans small RNA related to oxidation).

To determine whether DsrO and *msrA* may interact, we characterized the expression patterns of both genes under stress treatments using qRT-PCR. DsrO expression levels were quickly and significantly enhanced with increasing H_2_O_2_ concentration in the medium (≤80 mM). In the WT, DsrO was upregulated for up to 50 min in the 80 mM H_2_O_2_ treatment group (Fig. 3C). In contrast to *msrA*, DsrO could quickly respond to UV light (Fig. 3D), heat (Fig. 3E), and cold (Fig. 3F), indicating that DsrO participates in the regulation of several abiotic stress responses in D. radiodurans.

**FIG 4:**
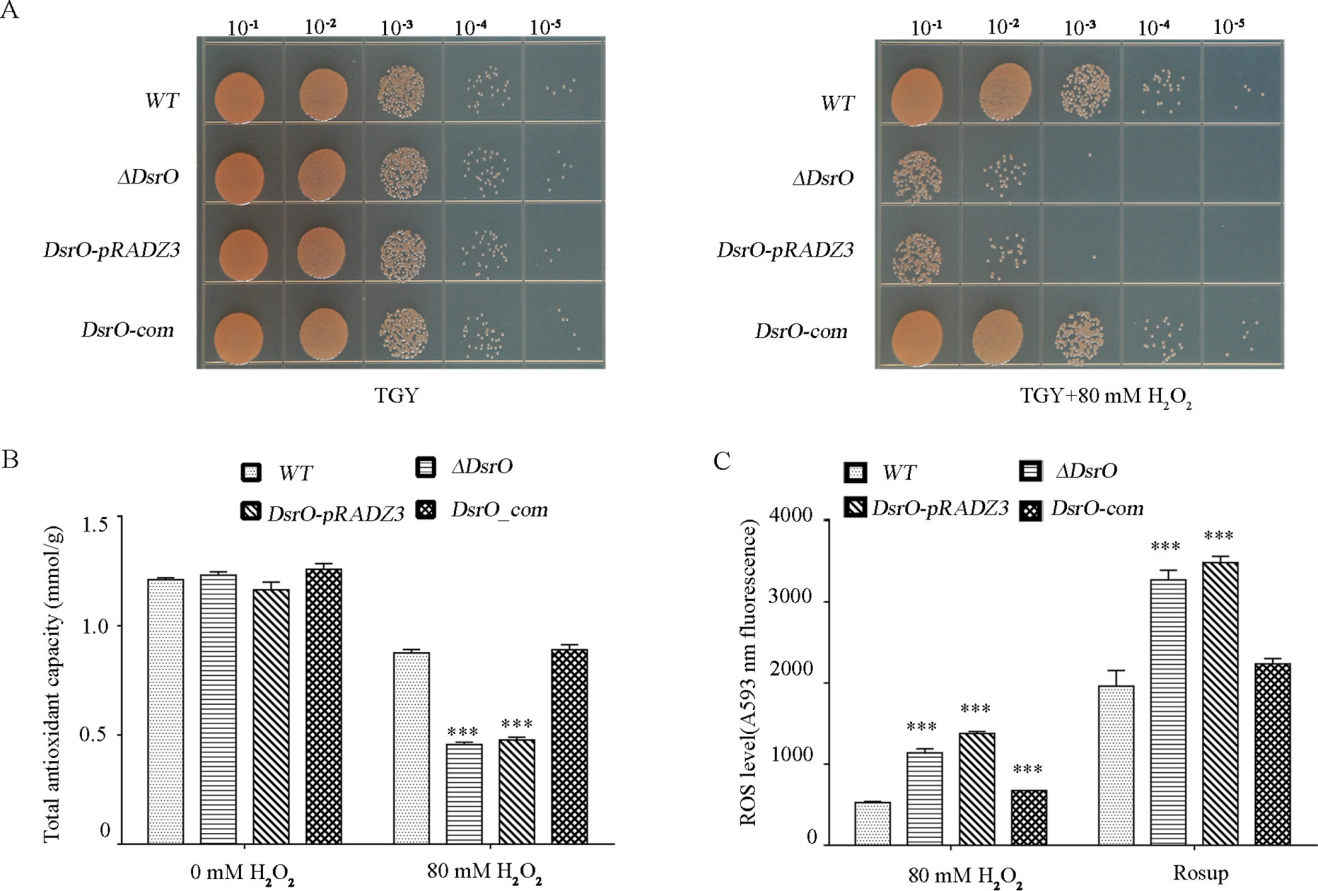
The DsrO knockout mutant is sensitive to oxidative stress. (A) Phenotypes of different strains under treatment with 0 mM H_2_O_2_ and 80 mM H_2_O_2_ for 30 min. The bacterial cells were serially diluted 10 times. (Left) untreated control; (right) 80 mM H_2_O_2_ treatment. WT, wild-type strain; ΔDsrO, DsrO-deleted mutant; DsrO-*pRADZ3*, the DsrO mutant with *pRADZ3* empty plasmid; DsrO-com, DsrO mutant supplemented with the DsrO gene. (B and C) Total antioxidant capacity and ROS level of WT, ΔDsrO, DsrO*-pRADZ3*, and DsrO-com. Rosup is a reactive oxygen species positive-control reagent. One-way ANOVA and Dunnett’s multiple-comparison test; ***, *P* ≤ 0.001; *, *P* ≤ 0.05. Experiments were performed at least three times, and the data are presented as the means ± SEM.

To characterize the physiological function of DsrO, we investigated the growth phenotypes of the WT, the mock strain (ΔDsrO*-pRADZ3*), the DsrO knockout strain (ΔDsrO), and the complementation strain under oxidative stress (Fig. 4). Under normal conditions (CK), there was no significant difference in growth between the tested strains. However, the DsrO mutant strain showed strong sensitivity to the 80 mM H_2_O_2_ treatment, whereas the complementation strain was nearly identical to the WT (Fig. 4A). Consistent with the experiments of *msrA* function analysis, significantly reduced total antioxidant capacity and greatly increased intracellular ROS levels were observed in the DsrO knockout compared to the WT (Fig. 4B and C). Contrary to our expectations, exposure to UV light, heat, or cold stress did not affect bacterial growth in any of the tested strains (Fig. S3). These observations showed that only oxidative stress influenced growth in the DsrO mutant strain, even though DsrO expression was significantly induced during several abiotic stress conditions (Fig. 3C to F). In conclusion, based on the above-described results, we propose that DsrO regulates the oxidative stress response of D. radiodurans by directly regulating *msrA* mRNA levels.

### DsrO directly stabilizes *msrA* expression by base pairing within the coding sequence.

To determine how DsrO interacts with its target, *msrA*, we performed 5′ RACE (rapid amplification 5′ end of full-length cDNA) analysis and confirmed that DsrO transcription started from the nucleotide of adenine and that the full length of the DsrO gene was 83 bp (Fig. S4). We further applied microscale thermophoresis (MST) to identify the binding strength between DsrO and *msrA* mRNA. The binding force curve indicated that DsrO could directly bind to *msrA* mRNA with a dissociation constant (*K_d_*) of 3.80 ± 2.77 μM (Fig. 3B). When all of the bases in the complementation sequence of DsrO or *msrA* mRNA were mutated, the binding force between DsrO and *msrA* mRNA disappeared (Fig. S5C), supporting that the interaction occurs between bases 66 and 77 in DsrO and 54 and 65 in *msrA*. To determine which nucleotide is critical for their interactions, we mutated each base in these regions of DsrO and *msrA* and tested the affinity strength (Fig. S5A). All of the binding capacity was lost when the first two bases (66 and 67) in DsrO were mutated (Fig. S6A and B); other point mutations changed the dissociation constant, and these changes were significant compared to the WT (Fig. S6C to F). For *msrA*, all binding capacity was lost when base 64 or 65 (corresponding to base 66 or 67 in DsrO) was mutated (Fig. S7A and B). As with DsrO, the effects of other point mutations on the dissociation constant were significant in comparison to the WT (Fig. S7C to F). In conclusion, the first two bases at the 5′ end of the DsrO ring are the key bases for the interaction between DsrO and *msrA* mRNA in D. radiodurans.

**FIG 5:**
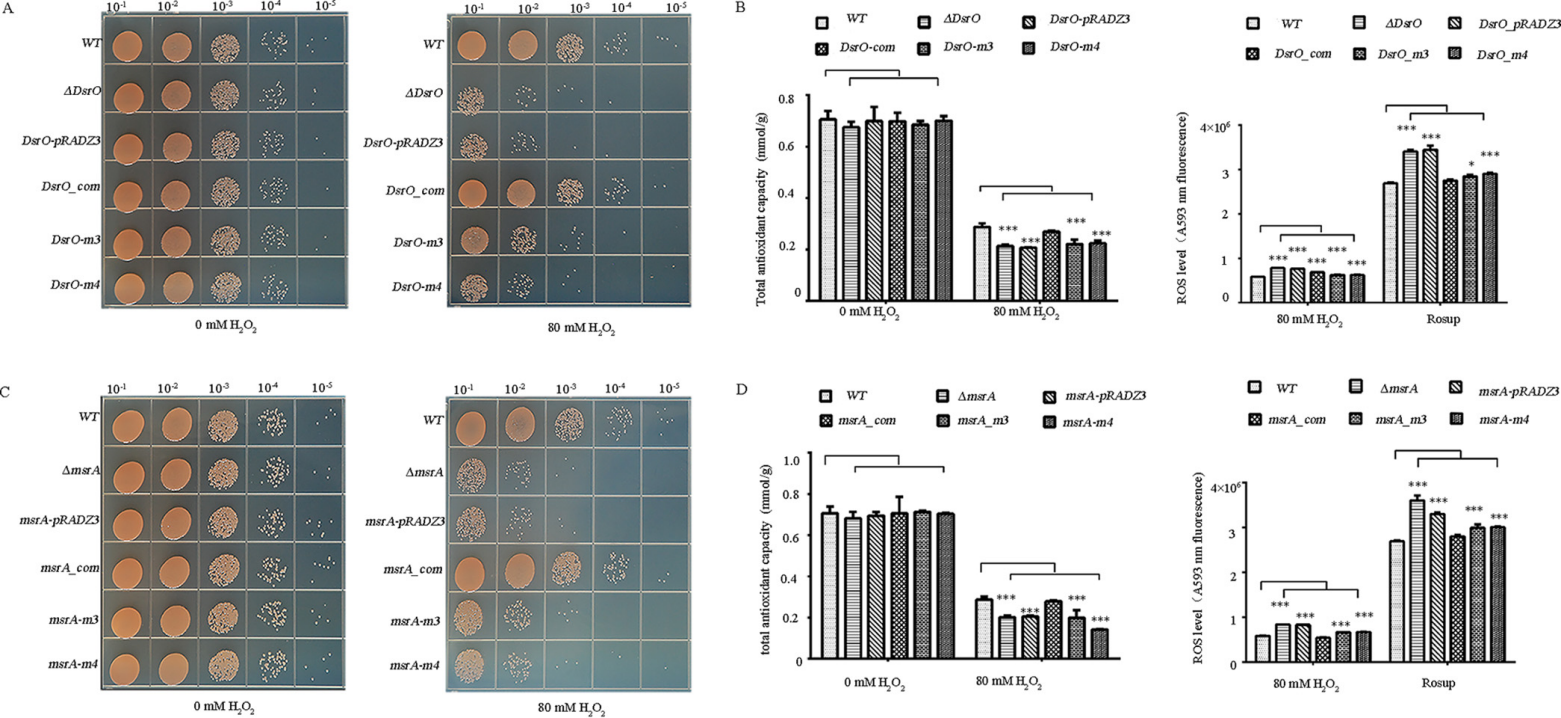
The key base mutation of the binding region between DsrO and *msrA* confers a phenotype sensitive to oxidative stress. (A and C) Survival of DsrO (A) or *msrA* (C) point-mutation strains upon exposure to 80 mM H_2_O_2_. (B and D) Total antioxidant capacity and ROS level of different DsrO (B) or *msrA* (D) point-mutant strains upon exposure to 80 mM H_2_O_2_. WT, wild-type strain; ΔDsrO/*msrA*, DsrO/*msrA*-deleted mutant; DsrO/*msrA-pRADZ3*, the DsrO/*msrA* mutant with *pRADZ3* empty plasmid; DsrO/*msrA*-com, DsrO mutant supplemented with DsrO/*msrA* gene; DsrO/*msrA*-m3, DsrO/*msrA*_com strain with the 1st mutated base, DsrO/*msrA*_m4, DsrO_com strain with the 2nd mutated base; Rosup is a reagent that acts as a positive control. Asterisks indicate statistically significant differences compared to untreated cells. One-way ANOVA and Dunnett’s multiple-comparison test; ***, *P* ≤ 0.001; *, *P* ≤ 0.05. Experiments were performed at least three times, and the data are presented as the means ± SEM.

To test the effects of these two bases on the physiological functions of D. radiodurans under oxidative stress, we investigated the growth phenotypes of the WT, mock strain (DsrO-pRADZ3), knockout mutant strain (DsrO-m3 or DsrO-m4), and complementation strain under oxidative stress (Fig. 5). Under normal growth conditions, there was no significant difference in growth between any of the tested strains; however, the DsrO mutant strains (DsrO-m3/m4) were significantly more sensitive to treatment with 80 mM H_2_O_2_, whereas the complementation strain was nearly identical to the WT (Fig. 5A). Significantly reduced total antioxidant capacity and strongly increased intracellular ROS levels were also observed in the DsrO-m3/m4 mutant strains in comparison to the WT (Fig. 5B). The effect of *msrA* point mutation on the oxidative tolerance of D. radiodurans was almost the same as that of DsrO; two mutations in *msrA* (at base 64 or 65, corresponding to base 66 and base 67 in DsrO) led to high oxidative stress sensitivity (Fig. 5C). These *msrA-m3* and *msrA-m4* mutants were sensitive to oxidative stress, with significantly decreased physiological indices (intracellular ROS levels, total antioxidant capacity) compared with the WT (Fig. 5D). We thus conclude that the first two bases located on the stem-loop structure of DsrO not only affect the binding strength between DsrO and *msrA* but also determine the level of tolerance to oxidative stress in D. radiodurans.

### *msrA* targeted by DsrO forms a stem-loop structure that is required for *msrA* mRNA stability and protein translation.

**FIG 6:**
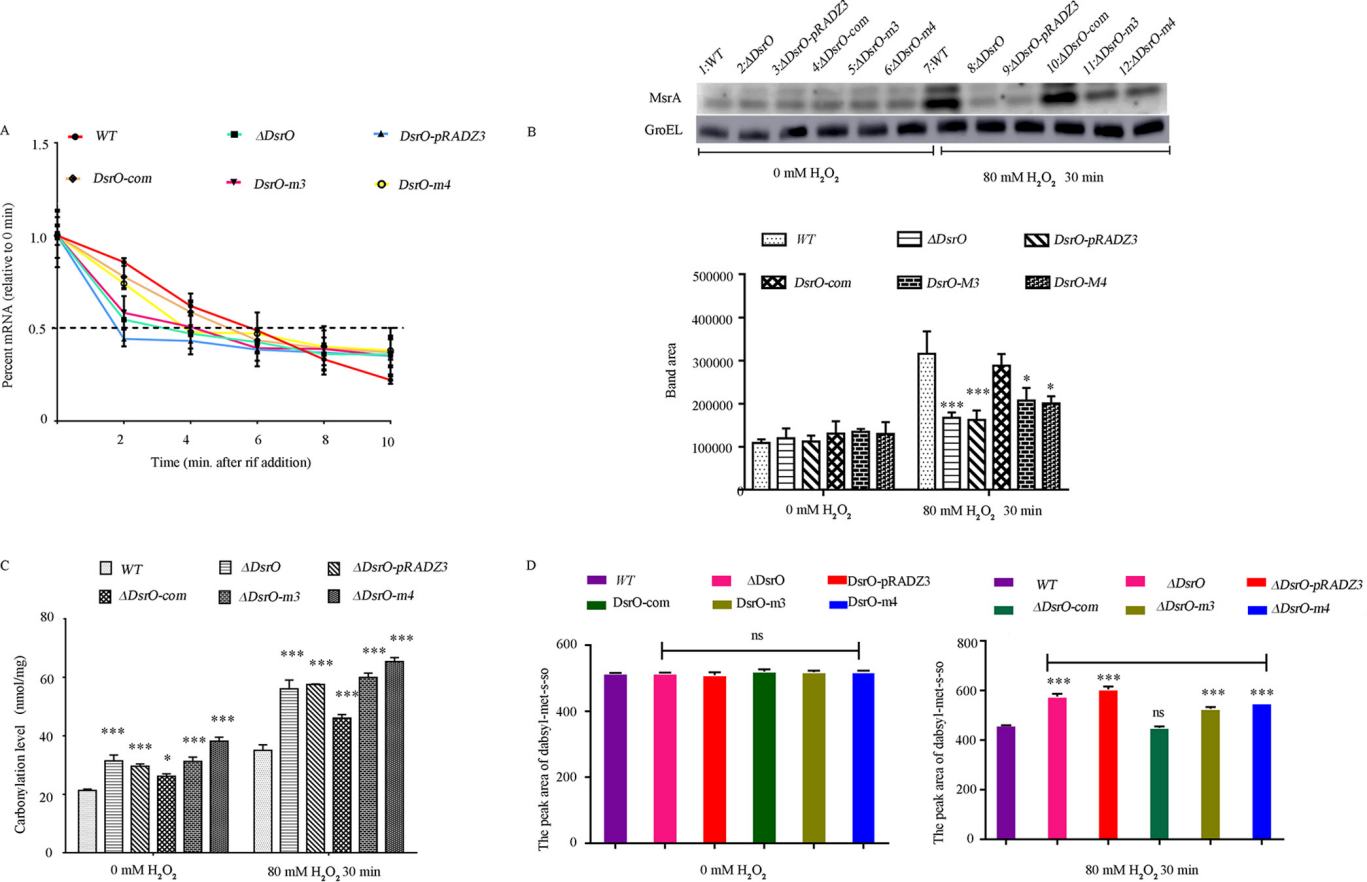
DsrO promotes the stability of *msrA* mRNA and enhances MsrA protein translation. (A) Time of *msrA* mRNA reaching its half-life in different strains. (B) Western blot of MsrA in different strains; bands of target protein (MsrA) and reference protein (GroEL) are indicated by arrows. WT, wild-type strain; ΔDsrO, DsrO deleted mutant; DsrO*-pRADZ3*, the DsrO mutant transformed with *pRADZ3* empty plasmid; DsrO-com, DsrO mutant supplemented with DsrO gene. (C) Intracellular carbonylation level and the percentage reduction of dabsyl-MetSO peak area in different samples. (D) The contents of intracellular Met-SO in DsrO mutants. One-way ANOVA and Dunnett’s multiple-comparison test; ***, *P* ≤ 0.001; *, *P* ≤ 0.05.

The sRNAs can have a direct effect on the stability or translation levels of target mRNAs (33, 34). We therefore analyzed the half-life of *msrA* mRNA in D. radiodurans R1 and the mutant strains (*msrA-m3* and *msrA-m4*). Analysis of mRNA stability showed that the *msrA* mRNA half-life was 5 min in the WT and only 2 min in the ΔDsrO and DsrO-m3/m4 mutant strains (Fig. 6A). Western blot and level of carbonylation results showed that the protein synthesis of MsrA was reduced in DsrO knockout and DsrO-m3/m4 point mutation strains in comparison with the WT, indicating that DsrO-m3/m4 point mutation likely affects protein translation (Fig. 6B and C). This indicated that DsrO and its key bases are essential for maintaining the stability of *msrA* mRNA.

MsrA can catalyze the reduction of methionine-S-sulfoxide (Met-S-SO) to methionine in proteins and free amino acids. We therefore used high-performance liquid chromatography (HPLC) to analyze the contents of intracellular Met-SO in DsrO mutants. The metabolites measured were dabsyl-Met-S-SO, dabsyl-Met-R-SO, and dabsyl-Met. In the ΔDsrO mutant lines (DsrO-m3, DsrO-m4; *msrA-m3*, *msrA-m4*), dabsyl-Met-S-SO content was reduced by 30% compared to the WT; complementation of DsrO in ΔDsrO recovered the levels to 97% of the WT (Fig. 6D). In summary, these results suggest that the sRNA DsrO directly stabilizes the target mRNA *msrA* to promote its translation under oxidative stress.

### *msrA* gene expression is transcriptionally regulated by the DrRRA transcription factor.

**FIG 7:**
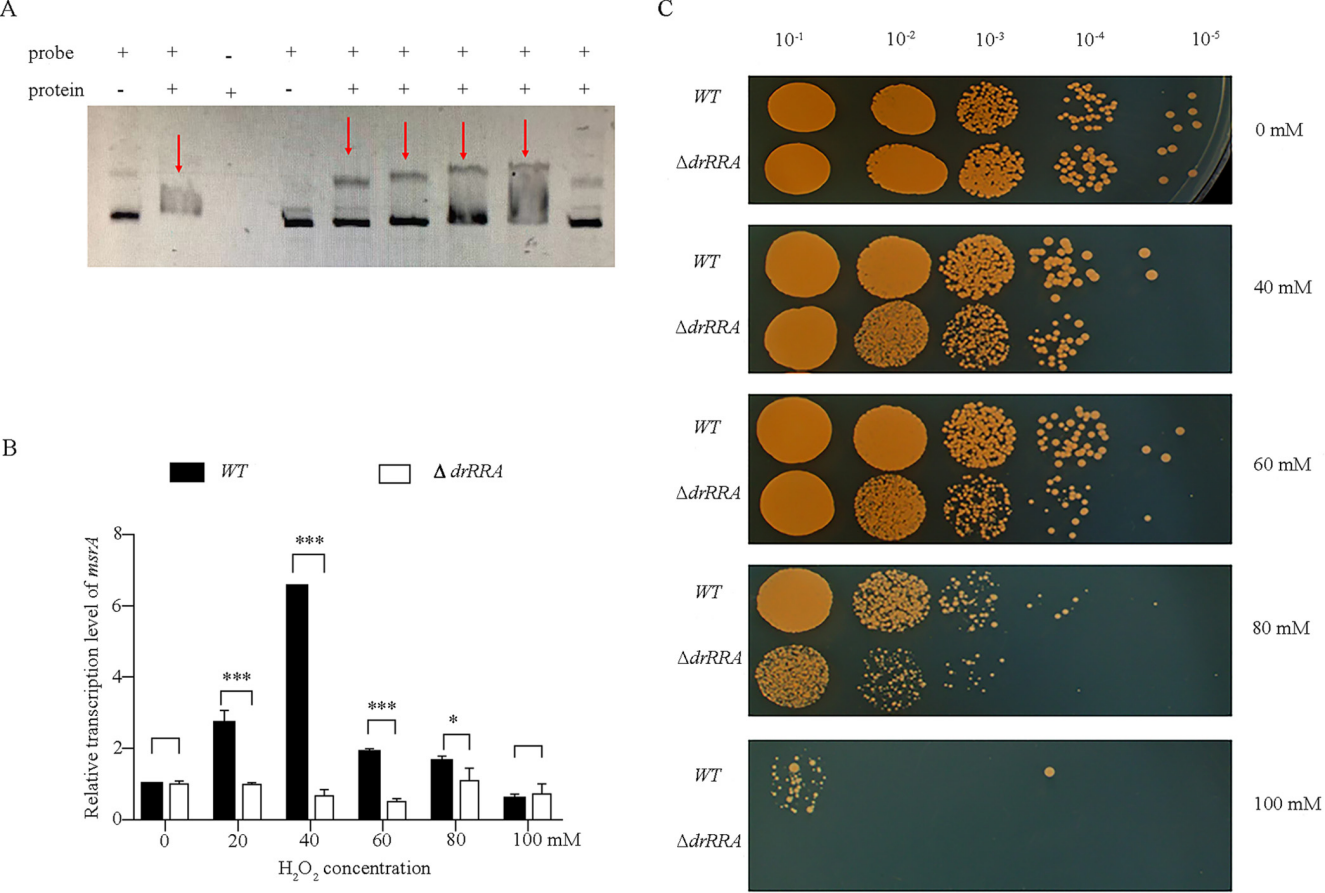
DrRRA is the transcriptional regulator of the *msrA* gene in D. radiodurans. (A) Electrophoretic mobility shift assay of DrRRA binding to the Cy5-labeled *msrA* promoter specifically. (Lane 1 [left] and Lane 2 [right]) Detection result of *dr_0997* promoter; lane 1 is the band of the single labeled *dr_0997* probe; lane 2 is the result of the labeled *dr_0997* probe with 6 μg DrRRA protein. Lanes 3 to 9) Detection result of *msrA* promoter; (lane 3) DrRRA protein; (lanes 4 to 8 reaction system contained 40 ng labeled *msrA* probe and 0 μg, 3 μg, 6 μg, 9 μg, and 12 μg DrRRA protein. (Lane 9) Reaction system containing 40 ng labeled *msrA* probe, 500 ng unlabeled *msrA* probe, and 6 μg DrRRA protein. (B) The deletion of the DrRRA gene in D. radiodurans resulted from the induction decrease of *msrA* expression activated by the oxidative stress. (C) H_2_O_2_ sensitivity assays of different DrRRA strains. Spotted agar plates after H_2_O_2_ treatment and serial dilution. Different strains were treated with 0 to 100 mM H_2_O_2_ for 30 min and dotted in the plates. WT, wild-type strain, Δ*drRRA*, *drRRA* deleted mutant. One-way ANOVA, Dunnett’s multiple-comparison test; ***, *P* ≤ 0.001; *, *P* ≤ 0.05. Experiments were performed at least three times, and data are presented as means ± SEM.

To identify the protein that transcriptionally regulates *msrA* in response to oxidative stress, we screened transcription factors coexpressed with *msrA* based on the transcriptomic data and a previously reported promoter analysis (35). DrRRA (Deinococcus radiodurans radio-resistance regulator) was predicted to bind to the *msrA* promoter based on previous transcriptomic data and interaction predictions. To confirm the interaction of DrRRA with the *msrA* promoter, we expressed and purified an N-terminally His-tagged DrRRA fusion protein from Escherichia coli and performed electrophoretic mobility shift assays (EMSAs). We observed a specific shift in mobility of the *msrA* promoter probe (500 bp) and a concomitant loss of the free *msrA* promoter probe, and stronger bands were detected with increasing concentrations of the DrRRA protein (Fig. 7A). In the DrRRA mutant cells, *msrA* expression was reduced, and no change occurred in response to oxidative stress (Fig. 7B). These data together indicate that DrRRA directly and specifically binds to the promoter region of *msrA*. Further analysis of the *drRRA* knockout line indicated that the deletion significantly decreased its tolerance to treatment with H_2_O_2_ (Fig. 7C), supporting that *msrA* is transcriptionally regulated by DrRRA.

## DISCUSSION

**FIG 8:**
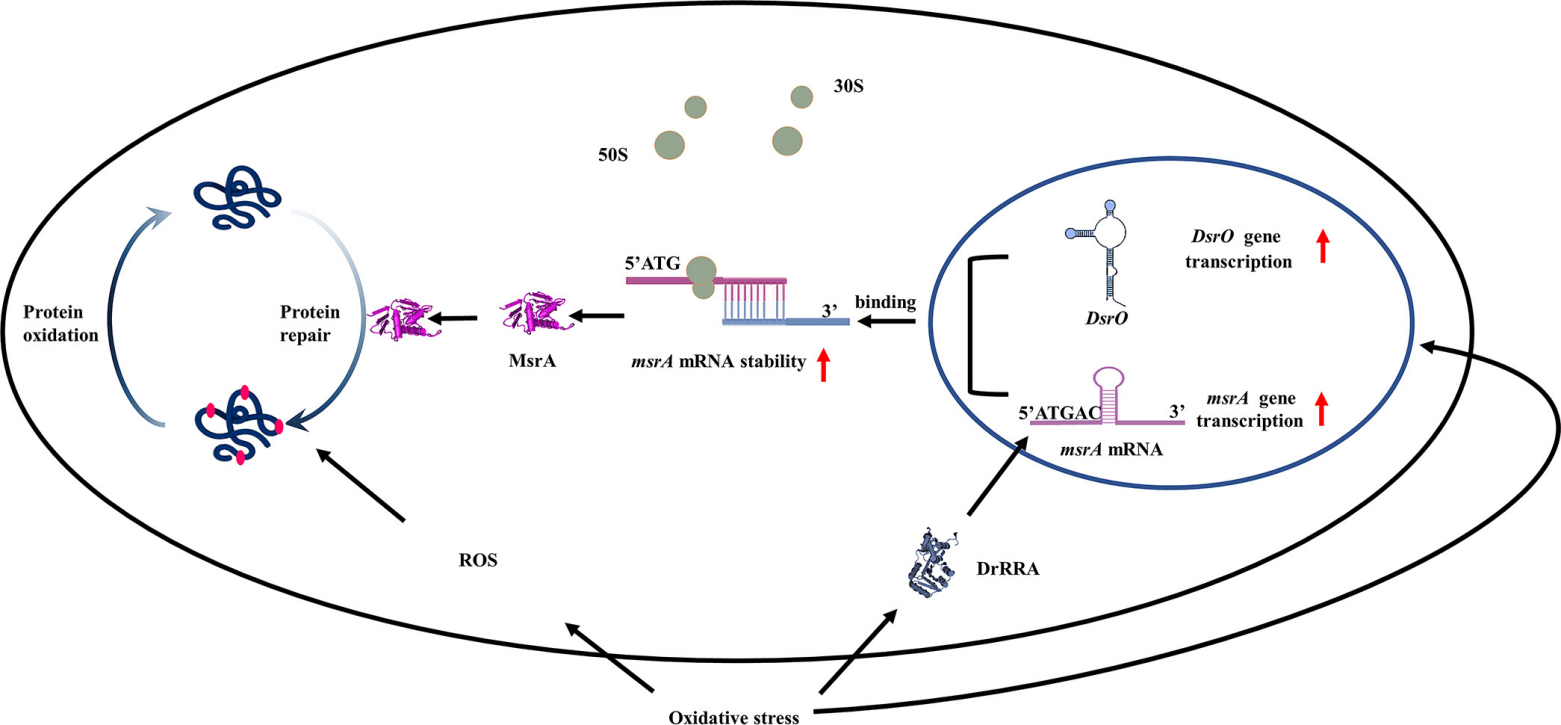
Diagram of DsrO posttranscriptionally regulating *msrA* in D. radiodurans. (i) D. radiodurans cells produce ROS under the treatment of exogenous H_2_O_2_ or oxidative stresses. ROS oxidize the methionine residue of proteins into methionine sulfoxide, inactivating the protein or causing cell death. (ii) When exogenous H_2_O_2_ enters the D. radiodurans cell, the sRNA DsrO and *msrA* are rapidly induced. At the same time, the transcription factor DrRRA upregulates the expression of *msrA* at the transcriptional level. (iii) DsrO binds to the stem-ring structure of *msrA* mRNA and thus enhances the stability of *msrA* mRNA and improves MsrA expression at the translational level. (iv) The increased MsrA reduces the methionine sulfoxide on the protein to methionine to restore protein function, thus promoting the oxidation resistance of D. radiodurans. The large gray-green circle represents the 50s large subunit of the ribosome, the small circle represents the 30 s small subunit of the ribosome, the blue stem-loop represents sRNA DsrO, the light purple stem-loop represents *msrA* mRNA, the gray fold represents DrRRA protein, and the bright purple fold represents MsrA protein. The dark blue folds represent proteins in the cell, and the rose dots above represent Met residues on the proteins.

To survive in extreme environments, D. radiodurans has developed efficient antioxidant systems to decrease ROS production, repair DNA damage, and recover the functions of oxidized proteins (36). Compared to the ROS-sensitive E. coli, D. radiodurans has highly active protein repair systems to mitigate ROS damage and decrease oxidized protein and protein carbonylation. Because the Msr protein is a typical enzyme with the ability to repair oxidized Met (37), we investigated the function of MsrA in D. radiodurans and analyzed its regulatory mechanisms. Here, we report the function of MsrA in D. radiodurans during oxidative stress and show that *msrA* is regulated by both the sRNA DsrO and the transcription factor DrRRA to quickly respond to oxidative stress, improving the antioxidant ability of D. radiodurans compared to ROS-sensitive bacteria (Fig. 8).

### MsrA in D. radiodurans R1 primarily functions under oxidative stress.

Different abiotic and biotic stresses can result from ROS accumulation and cell damage in bacteria (38). Methionine, as an initiator amino acid residue, is fundamental for protein translation and ranks as the most sensitive to oxidation (39, 40). To restore the function of the damaged protein, *msrA* is strongly upregulated in response to high radiation, desiccation, or low temperature (41). In D. radiodurans, we also found that *msrA* expression was significantly enhanced when the cells were exposed to 80 mM H_2_O_2_, UV radiation, or temperature stress (Fig. 1, Fig. S1). In D. radiodurans cells exposed to 60 mM H_2_O_2_ for 30 min, the maximum transcription level of *msrA* was almost 13-fold higher than that in the control group (Fig. 1A). *msrA* knockout D. radiodurans cells were susceptible to H_2_O_2_ damage (Fig. 2), and the *msrA* mutant had a deficiency in antioxidative capacity and increased levels of dabsyl-Met-S-SO. These results were consistent with the modification function of MsrA protein previously observed in other organisms (7).

Contrary to our expectations, *msrA* knockouts were not sensitive to UV radiation or heat/cold stress, even though *msrA* was strongly upregulated in all three stress conditions in D. radiodurans (Fig. S2). These results indicate that MsrA is a key protein in the response to H_2_O_2_ treatment but does not function during UV radiation or temperature stress. Another possibility is that a reduction in the level of damaged proteins requires the participation of both MsrA and MsrB under UV radiation or hot/cold stress. Similar to most other organisms, the D. radiodurans genome contains one copy each of *msrA* and *msrB* (42). Based on their primary sequence and protein structures, MsrA and MsrB have no significant homology. MsrA and MsrB possess the ability to reverse S,R-MetSO forms into Met (43). Molecular analysis indicated that *msrA* and *msrB* have different expression patterns and physiological roles when bacterial cells are exposed to various abiotic stresses, such as iron limitation (44). Consequently, future work will aim to functionally analyze MsrB and its connection with MsrA in protein repair under UV radiation or hot/cold stress.

### *msrA* mRNA stability is crucial for the antioxidant capacity of D. radiodurans.

The sRNAs typically bind target mRNAs through complementation, and this binding alters mRNA stability and translation (45, 46). Using sRNA target mRNA analysis and molecular interaction analysis, we demonstrated that the sRNA DsrO binds to the coding sequence region of *msrA* mRNA *in vitro* (Fig. 3). Site-directed mutagenesis of the complementary sequence between DsrO and *msrA* demonstrated that the 1st and 2nd bases of DsrO are crucial for the stability of *msrA* mRNA (Fig. S6 and S7). As expected, viability was decreased in DsrO and *msrA* knockouts and point mutants when treated with 80 mM H_2_O_2_ (Fig. 5). We thus propose that DsrO is a positive regulator of *msrA* mRNA stability, and this improved mRNA stability benefits MsrA protein translation, ultimately enhancing MsrA enzyme activity (Fig. 6). Bacterial sRNAs often negatively regulate transcription by binding the mRNA of a target gene and enhancing transcription termination (34). Contrary to the regulatory pattern of most investigated bacterial sRNAs, DsrO expression accelerated the translation of *msrA*, as indicated by Western blotting (Fig. 6). One possible explanation is that the interaction between DsrO and *msrA* mRNA forms a secondary structure to activate translation. This regulatory mechanism is similar to the activation of translation *fepA* mRNA by the binding sRNAs OmrA and OmrB in E. coli (47).

D. radiodurans has an unusual capability to repair extensive DNA damage, maintaining genome stability under abiotic stress conditions, including high gamma and UV radiation, hydrogen peroxide exposure, and desiccation (48). The efficient DNA repair system in D. radiodurans requires the participation of RecA, PprA, and DNA glucoamylase (49). Under high H_2_O_2_ levels, the Mets in E. coli RecA may be oxidized to MetSO, losing function. MsrA/B are required for maintaining the recombinase function of RecA under oxidative stress (50). These findings imply that the regulation of oxidized protein repair involves cross talk with the DNA repair signaling pathway in D. radiodurans. In this study, we demonstrated that DrRRA transcriptionally regulates *msrA* expression by directly binding to its promoter (−73 bp to −54 bp: CTCCCGGCGCCTGTCCCCTC). DrRRA has a regulatory role in multiple antioxidation and DNA repair pathways for preserving DNA in D. radiodurans (35). The deletion of *drRRA* results in decreased viability under oxidative stress as well as the delay of genome recovery, indicating that DrRRA and DsrO may jointly regulate DNA and protein modification. Because *msrA* expression is regulated by DrRRA at the transcriptional level and by DsrO at the posttranscriptional level, future work should focus on determining whether other proteins/factors integrate the regulatory activities of DrRRA and DsrO in D. radiodurans.

## MATERIALS AND METHODS

### Bacterial strains and plasmids.

The liquid growth media used were TGY broth (1% tryptone, 0.1% glucose, 0.5% yeast extract, pH 7.0) and LB broth (1% tryptone, 1% yeast extract, 0.5% NaCl, pH 7.0). The solid growth media used were TGY and LB agar (TGY or LB broth with 1.5% agar). Deinococcus radiodurans R1 and its derivatives were grown in or on TGY with appropriate antibiotics (Table 1) at 30°C. Escherichia coli was grown in or on LB with appropriate antibiotics (Table 1) at 37°C. All liquid cultures were incubated with shaking at 220 rpm. The plasmids and strains used in this study are listed in Table 1.

**TABLE 1:** The list of plasmids and strains

Plasmid/strain	Description	Source
pRADZ3	Shuttle vector for E. coli and D. radiodurans, Chl^r^ (D. radiodurans), Amp^r^ (E. coli)	Laboratory stock
pKatAPH3	To amplify the kanamycin resistance gene	Laboratory stock
pKatAAD2	To amplify the spectinomycin resistance gene	Laboratory stock
D. radiodurans *R1*	Wild-type, served as the strain for generating the mutants	Laboratory stock
E. coli DH5α	The strain for expressing the shuttle plasmid pRADZ3	CW Biotech
Δ*msrA* mutant	D. radiodurans with genomic deletion of *msrA* gene	This study
*msrA*-*pRADZ3*	*msrA* mutant with pRADZ3 shuttle plasmid introduced into its genome	This study
*msrA_com*	Complementation of the *msrA* deletion in D. radiodurans, transformation of *msrA* mutant with pRADZ3 plasmid expressing D. radiodurans *msrA* gene	This study
*msrA_m3*	*msrA* complementary strain with a mutated base which is the first base in the binding sequence	This study
*msrA_m4*	*msrA* complementary strain with a mutated base which is the second base in the binding sequence	This study
ΔDsrO mutant	D. radiodurans with genomic deletion of DsrO gene	This study
DsrO_pRADZ3	DsrO mutant with pRADZ3 shuttle plasmid introduced into its genome	This study
DsrO_com	Complementation of the DsrO deletion in D. radiodurans, transformation of DsrO mutant with pRADZ3 plasmid expressing D. radiodurans DsrO gene	This study
DsrO_m3	DsrO complementary strain with a mutated base which is the first base in the binding sequence	This study
DsrO_m4	DsrO complementary strain with a mutated base which is the first base in the binding sequence	This study

To generate the *msrA* and DsrO mutants, sequences 1 kb upstream (U) and 1 kb downstream (D) of the gene and the spectinomycin resistance gene (S) were amplified separately. Then, the three fragments were assembled in a molar ratio of 1:1:1 under the catalysis of homologous recombinase. Finally, the assembled product was used as a template, and P1-F/P3-R was used as a primer to amplify the fusion fragment USD. Finally, the fused fragment was introduced into D. radiodurans as previously reported (51). For complementation of the *msrA* gene in D. radiodurans, *msrA* and the associated promoter were inserted into the pRADZ3 plasmid between the endonuclease BamHI and SpeI sites, and then the recombinant plasmid was transformed into D. radiodurans R1.

### H_2_O_2_ stress treatment and oxidative tolerance phenotype analysis.

D. radiodurans strain R1 and its derivatives were grown in TGY broth until the beginning of the logarithmic growth phase (optical density at 600 nm [OD_600_], 0.5 to 0.8) and then transferred into fresh TGY broth with or without H_2_O_2_ (final concentration, 80 mM) for 30 min. For oxidative tolerance analysis of the different strains, 100 μL of bacterial culture was added to 900 μL of phosphate-buffered saline (PBS). Cultures were serially diluted 10-fold to a final concentration of 10^−5^. The diluted culture (7 μL) was dropped vertically onto TGY plates in the order of concentration from 10^−1^ to 10^−5^. The plates were incubated at 30°C for 3 days, and growth phenotypes were recorded.

### Total RNA extraction and Northern blotting.

Total RNA extraction was performed following the protocol of the PureLink RNA minikit (Invitrogen, California, USA). For Northern blotting, 10 μg of total RNA from different samples was separated on a 6% urea gel (National Diagnostics, Georgia, USA) for 1 to 1.5 h at 120 V. After electrophoresis, the RNA was transferred to a nylon membrane using semidry transfer blotting. The membrane was incubated at 60°C for 1 to 2 h to facilitate cross-linking to the EDC (1-ethyl-3-[3-dimethylaminopropyl]carbodiimide) reagent.

The hybridization probes were labeled following the instructions of the Pierce RNA 3′ end biotinylation kit (Thermo Fisher Scientific), and then the biotin-labeled probes were hybridized with the membrane at 37°C overnight in a hybridization oven. The nylon membranes were washed twice with stringent buffer, and the hybridization signals were detected following the procedure of the chemiluminescent nucleic acid detection module (Thermo Fisher Scientific).

### Rapid amplification of the 5′ end (RACE) of the DsrO gene.

The 5′/3′ RACE kit 2nd generation (Roche, Mannheim, Germany) was used to amplify the end of the DsrO gene. The primers were designed according to the genome sequence of the D. radiodurans R1 strain. The full-length cDNA of the gene was amplified after cDNA synthesis (cDNA synthesis buffer, 4 μL; deoxynucleotide mixture, 2 μL; primer SP1, 1 μL; total RNA, 1 μg; transcriptor reverse transcriptase, 1 μL; and double-distilled water _[ddH2O]_, up to 20 μL) and 2 cycles of nested PCR (dA-tailed cDNA, 5 μL; oligonucleotide dT-anchor primer, 1 μL; primer SP2, 1 μL; deoxynucleotide mixture, 1 μL; expanded high-fidelity buffer, 5 μL; double-distilled water, up to 50 μL).

### Real-time quantitative PCR (qRT-PCR).

Gene expression was analyzed with qRT-PCR. First-strand cDNA synthesis was performed with the PrimeScript RT reagent kit (TaKaRa Biomedical Technology, Beijing, China). First, 2 μL 5× genomic DNA (gDNA) eraser buffer, 1 μL gDNA eraser, and 1 μg total RNA were incubated at 42°C for 2 min to remove genomic DNA; then we added 1 μL PrimeScript RT enzyme mix I, 4 μL RT primer mix, 4 μL 5× PrimeScript buffer 2, and 1 μL RNase-free distilled water (dH_2_O) into the reaction liquid. qRT-PCR was performed with SYBR qPCR master mix (Vazyme, Nanjing, China) following the manufacturer’s instructions. The reaction system included 10 μL Hiscript III RT supermix, 1 μL RT-F primer, 1 μL RT-R primer, and 8 μL ddH_2_O, and the 16S rRNA gene was used as the internal control. The 2^–ΔΔ^*^CT^* values were calculated to indicate the expression levels of the target genes (52).

### Bioinformatics analysis.

To predict the binding sequence of DsrO in the *msrA* gene, IntaRNA software was used (http://rna.informatik.uni-freiburg.de/IntaRNA/Input.jsp) (53). The minimum binding nucleotide was set to 7. The secondary structures of *msrA* mRNA and DsrO were predicted with RNAalifold (http://rna.tbi.univie.ac.at/cgi-bin/RNAWebSuite/RNAalifold.cgi) (54). The fold algorithms and basic options used were minimum free energy (MFE), partition function, and the avoidance of isolated base pairs.

### Microscale thermophoresis analysis.

Full-length sRNA was synthesized by *in vitro* transcription; 5′ fluorescein amidite (FAM)-labeled mRNA was purchased from Shanghai GenePharma (Shanghai, China). The labeled mRNAs were then diluted to a fluorescence intensity of 200 to 1,000 according to the protocol in the user manual of the Monolith NT.115 system (NanoTemper Technologies, Munich, Germany). The detailed procedure was followed as previously reported (55, 56).

### Total antioxidant capacity, reactive oxygen (ROS) level, and intracellular carbonylation level analysis.

Bacterial ROS levels were measured with a ROS assay kit (Beyotime, Shanghai, China) containing the 2′,7′-dichlorodihydrofluorescein diacetate (DCFH-DA) fluorescent probe according to the manufacturer’s protocol (57). Bacteria loaded with the DCFH-DA probe were cultured to the initial logarithmic growth phase. Bacteria were collected by centrifugation and washed three times with TGY medium to remove probe contamination. Bacteria were then treated with H_2_O_2_ (final concentration, 80 mM) for 30 min. Rosup was used as the positive control as described in the kit brochure. Fluorescence intensity was analyzed at 488 nm excitation and 525 nm emission wavelengths.

Ferric reducing ability of plasma (FRAP) was performed to analyze the antioxidant capacity of bacteria using a total antioxidant capacity assay kit (Beyotime, Shanghai, China) (58). Cells were collected for ultrasonication at the beginning of the logarithmic growth phase. The bacteria were then centrifuged at 1,150 × *g* for 10 min. The supernatant (200 μL) was transferred to a new Eppendorf tube, and 180 μL of FRAP buffer was added. The mixture was incubated at 37°C for 3 to 5 min, and then the OD was measured at 593 nm.

A protein carbonyl colorimetric assay kit (Cayman Chemical, Ann Arbor, MI, USA) (59) was used to measure the protein carbonyl content of cell lysates in 96-well plates. Bacteria at the beginning of the logarithmic stage were collected for ultrasonication. The bacteria were then centrifuged at 3,000 rpm for 10 min. The supernatant (10 μL) was transferred to new plates and diluted 10 times. The amount of protein was calculated from a bovine serum albumin (BSA) standard curve (0.25 to 1.0 mg/mL). The amount of protein-hydrozone produced was quantified spectrophotometrically at an absorbance between 360 and 380 nm.

### Stability measurement of *msrA* mRNA.

Deinococcus radiodurans R1 and its derivatives were cultured to the initial logarithmic growth phase. Bacteria were treated with H_2_O_2_ (to a final concentration of 80 mM) for 30 min before rifampin (400 μg/mL) was added to stop gene transcription. At different time points after rifampin treatment (0 min, 2 min, 4 min, 6 min, 8 min, and 10 min), 2 mL of culture was collected from each sample. Total RNA was extracted as described above, and the level of *msrA* mRNA was detected with a real-time fluorescence quantification kit (Invitrogen).

### Western blot analysis.

Total protein was extracted from the H_2_O_2_-stressed and untreated bacteria as described above. The total protein (10 μg) of each sample was denatured at 100°C for 5 min and separated by 12% SDS-PAGE at 4°C for 1.5 h. After electrophoresis, the separated proteins were transferred from the gel onto a polyvinylidene difluoride (PVDF) membrane with a semidry electroblotter. The membrane was blocked with skimmed milk for 1 h. After that, the membranes were hybridized overnight with the primary anti-rabbit MsrA antibody (1:1,000). The primary polyclonal antibody against MsrA was produced by Genscript Biotechnology Company based on the MsrA protein sequence (Genscript, Nanjing, China). Next, the membrane was washed three times and treated with 1:5,000 goat anti-rabbit horseradish peroxidase (HRP) secondary antibody. Detection of the MsrA band was performed using the SuperSignal West Femto monoclonal kit (Thermo Fisher Scientific). The GroEL protein was used as the reference protein.

### Enzymatic activity analysis of MsrA.

MsrA enzymatic activity was analyzed as previously described (60). Dabsyl-Met (50 mM) was oxidized overnight by 500 mM H_2_O_2_, the supernatant was filtered by a C_18_ column, and finally, Dabsyl-MetSO was obtained when the column was eluted with acetonitrile. The reaction solution and the termination solution were prepared according to previous reports (60). Total protein extracts from different samples were added to the reaction solution. The reaction was terminated after incubation at 37°C for 1 h, and then the samples were centrifuged at 12,000 × *g* for 30 min at 4°C. The supernatant was used for HPLC. HPLC parameters and program settings all followed the procedure of the report of Vieira Dos Santos et al. (60).

### Electrophoretic mobility shift assay (EMSA).

His-tagged DrRRA was expressed using the *pET30a* (+) plasmid in E. coli strain BL21 and purified with His-tagged beads (Invitrogen, USA). The purified proteins were used in EMSA. EMSAs were performed according to the manufacturer’s instructions (Odyssey Infrared EMSA kit, LI-COR). DNA fragments used for Cy5-labeled probes were amplified by PCR with the labeled primers (Table S1). The Cy5-labeled DNA probe (40 ng) was incubated with different quantities of DrRRA protein and a positive *dr_0997* probe at 30°C for 30 min in binding buffer (20 μL). After incubation for 30 min, the solution, including protein-bound and free DNA, was separated by electrophoresis on 4.0% native polyacrylamide gels with 0.5× TBE buffer (44.5 mM Tris-HCl, 44.5 mM boric acid, and 1 mM EDTA, pH 8.0) at 100 V and 4°C. The gel was detected by an Amersham Imager 600.

### Data availability.

The genome sequence of Deinococcus radiodurans R1 has been deposited in NCBI GenBank under accession numbers CP015081 (https://www.ncbi.nlm.nih.gov/genome/?term=CP015081)(new) and AE000513 (https://www.ncbi.nlm.nih.gov/genome/?term=AE000513) (old). The accession numbers of the D. radiodurans genes used in this study are as follows: *msrA* (A2G07_04355 and DR_1849) and DR_0997 (A2G07_08515). The primers used in this study are listed in Table 2. Other relevant data are available within this article and its associated supplemental material.

**TABLE 2:** Primers used in this study

Primer name	Sequence (5′–3′)^*a*^	Purpose
P1-F	TAGTCCTGCACCCACTTCAGGGCCG	Δ*msrA* construct
P1-R	TCGGTCTCCATGCGAGCTCGAATTCGATAGGGACCGGAAAGCGGATTTAA	
P2-F	ATCCGCTTTCCGGTCCCTATCGAATTCGAGCTCGCATGGAGACCGA	
P2-R	TCGGTCTGCCACAGCACCCGCATTATTTGCCGACTACCTTGGTGAT	
P3-F	ACCAAGGTAGTCGGCAAATAATGCGGGTGCTGTGGCAGACCGAGGC	
P3-R	GCTCGCACAGCGTGGTGTATCTGGC	
P4-F	TCCAGCACACTGGCGGCCGTTACTAGTTCAGGAACCAGCCGTCGTCA	*msrA*_com construct
P4-R	GCATGCCTGCAGGTCGAATCGGATCCGCTCGGTGAAGACCCGGGGG	
P5-F	CACCGCTTAACATCACCCCT	ΔDsrO construct
P5-R	TGCTCGATGAGTTTTTCTAAGGATAACTTTGTTTTACGAGCGTT	
P6-F	CGTAAAACAAAGTTATCCTTAGAAAAACTCATCGAGCATCAAATG	
P6-R	CCTGGTTCAAACAACGAAAACATGGAGACCGAGGGCCCTTGACA	
P7-F	AAGGGCCCTCGGTCTCCATGTTTTCGTTGTTTGAACCAGGTGCA	
P7-R	GGGGATGAACGGCATTCTCT	
P8-F	TCCAGCACACTGGCGGCCGTTACTAGTCCACCCTCCCCGACGCCCTGCACG	DsrO_com construct
P8-R	GCATGCCTGCAGGTCGAATCGGATCCCGCCGTCCAGCAAACAAAAAACG	
*msrA* RT-F	CGCCGCACAATAGCAGCATG	qRT-PCR for *msrA* expression pattern
*msrA* RT-R	AGCGGTAATCGGGATTGGGCA	
DsrO RT-F	ACAGGTCTACGTTTCACTGTGGATA	qRT-PCR for *msrA* expression pattern
DsrO RT-R	CGCCGTCCAGCAAACAAAAAACGCT	
16s RT-F	ATTCCTGGTGTAGCGGTG	qRT-PCR for 16s rRNA
16s RT-R	ATTCCTGGTGTAGCGGTG	
*msrA SP1*	TCAGCGGAGTTTGTCGCCGTAGTACTGGCGCAGCT	5′ RACE for *msrA*
*msrA SP2*	CCGAGCTGCTCGATCATTTCGCGGGCCGTCTGTTC	
*msrA SP3*	AGCGGTAATCGGGATTGGGCACCGTACCGCCGATG	
DsrO *SP1*	CGCCGTCCAGCAAACAAAAAACGCTGAACAGTCAT	5′ RACE for DsrO
DsrO *SP2*	CAGCAAACAAAAAACGCTGAACAGTCATGCTTGAAAATCCGC	
DsrO *SP3*	AAAAACGCTGAACAGTCATGCTTGAAAATCCGCTTCAAAAGTT	

a The underlining indicates the restriction enzyme cutting site of Spe1(P4/P8-F) and BamHI(P4/P8-R).
