# Supplementary material for: A Novel Small RNA, DsrO, in Deinococcus radiodurans Promotes Methionine Sulfoxide Reductase (msrA) Expression for Oxidative Stress Adaptation
Source: Appl Environ Microbiol. 2022 May 16;88(11):e00038-22. doi: 10.1128/aem.00038-22 (PMC9195949; doi:10.1128/aem.00038-22)
Supplement: Supplemental file 1 — Fig. S1 to S7. Download aem.00038-22-s0001.pdf, PDF file, 0.7 MB [file aem.00038-22-s0001.pdf]

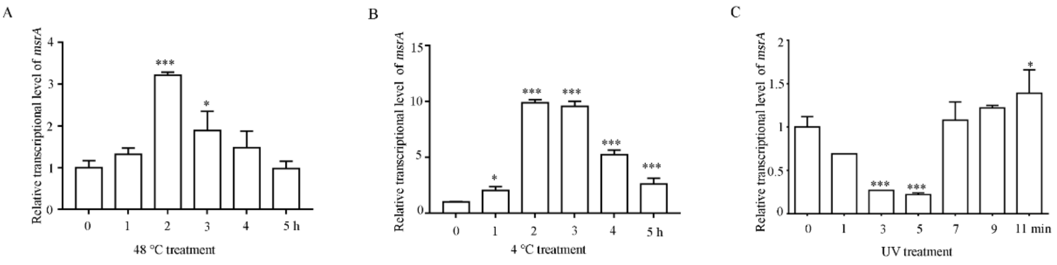

Fig. S1 *msrA* expression patterns under the treatments of UV radiation, heat (48°C) and cold (4°C) in *D. radiodurans*.

A-C: Relative expression levels of *msrA* in response to the treatments of heat (48°C) (A), cold (4°C) (B) and UV radiation (C) in *D. radiodurans*. Asterisks indicate statistically significant difference of the value compared to that of untreated cells (one-way Anova, Dunnett's multiple comparisons test; '***' means $P \leq 0.001$, '*' means $P \leq 0.05$). Experiments were performed at least three times, and data were presented as means SEM.

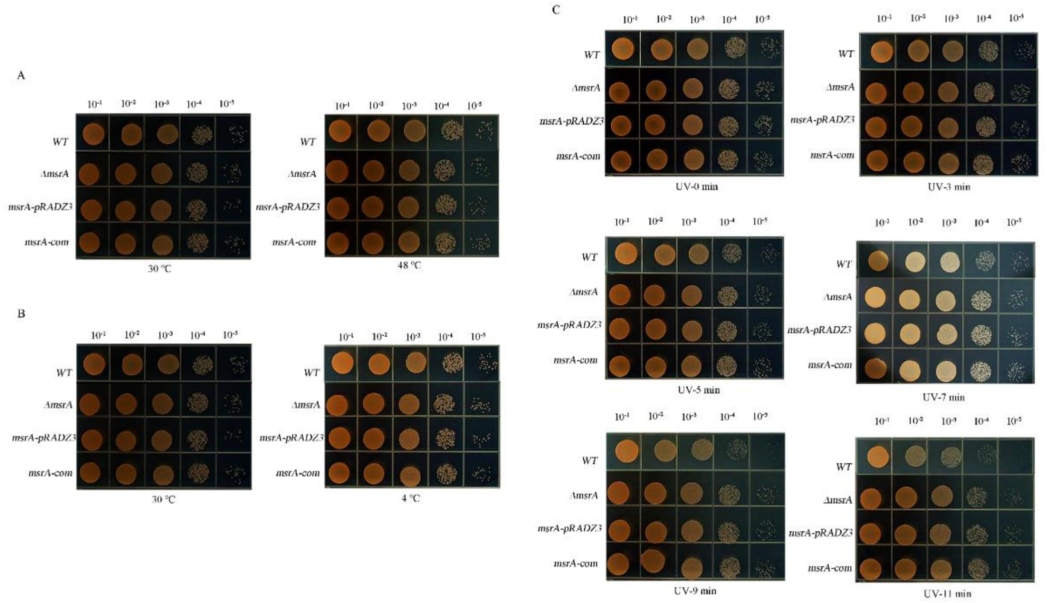

FIG S2 The knockout of *msrA* gene in *D. radiodurans* has no effect on the tolerance to UV radiation, heat (48℃) and cold (4℃) treatments in *D. radiodurans*.

A-C: Phenotype of different *D. radiodurans* strains under the treatments of UV radiation, heat (48°C) (A), cold (4°C) (B) and UV radiation (C).

Left images: untreated control; Right images: different abiotic stress treatments. *WT*: wild type strain, Δ*msrA*: *msrA* deleted mutant; *msrA-pRADZ3*: the *msrA* mutant transformed with *pRADZ3* empty plasmid; *msrA-com*: *msrA* mutant supplemented with *msrA* gene.

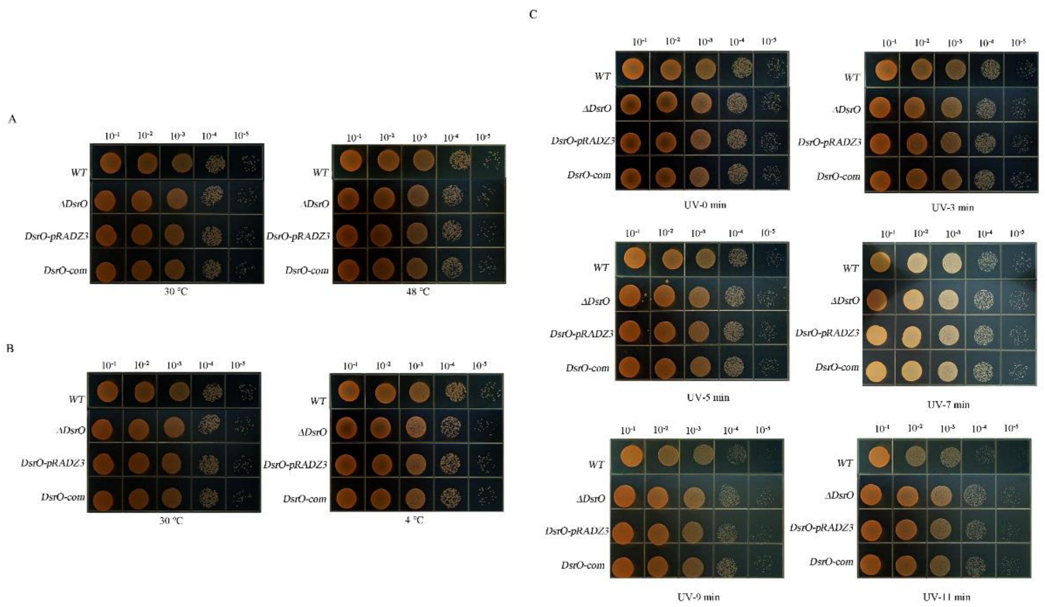

FIG S3 The knockout of *DsrO* gene in *D. radiodurans* has no effect on the tolerance to UV radiation, heat (48°C) and cold (4°C) treatments in *D. radiodurans*.

A-C: Phenotype of different *D. radiodurans* strains under the treatments of heat (48°C) (A), cold (4°C) (B) and UV radiation (C).

Left images: untreated control; Right images: different abiotic stresses. *WT*: wild type strain, Δ *DsrO*: *DsrO* deleted mutant, *DsrO-pRADZ3*: the *DsrO* mutant transformed with *pRADZ3* empty plasmid; *DsrO -com*: *DsrO* mutant supplemented with *DsrO* gene.

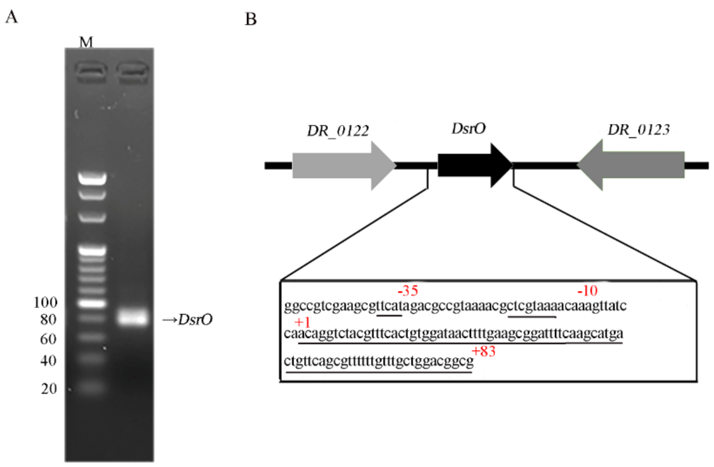

FIG S4 The characterization of *DsrO* and its gene locus.

A: The amplification result of *DsrO* gene using 5' rapid amplification of cDNA end (5'-RACE). M: 100bp DNA ladder; B: Physical map and nucleotide sequence of *DsrO*. Promoter elements (-35 and -10 box) are underlined; Transcription start site mapped by 5'-RACE is emphasized with arrows; *DsrO* nucleotide sequence is underlined from the start point.

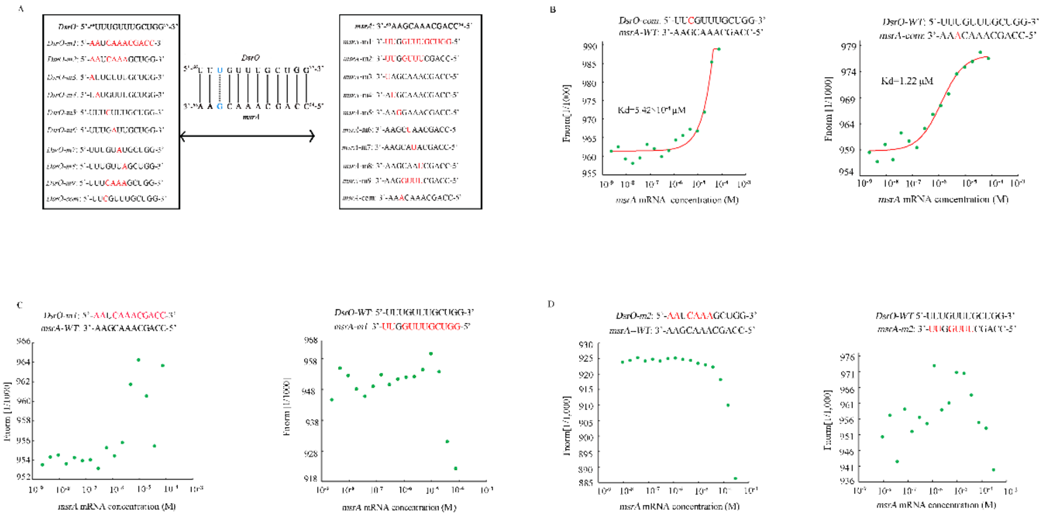

FIG S5 MicroScale Thermophoresis (MST) analysis the nucleotides affecting the affinity between *msrA* and *DsrO.*

A. The sketch map describing the continuous base mutation in *msrA* or *DsrO*. B. The mutation of non-complementary bases enhanced the affinity between *msrA* and *DsrO*. C-D. The completed and partial mutations of complementary sequences resulted from the lose of interacting between *msrA* and *DsrO*.

The K_d coefficients were determined employing the standard data analysis of MST with affinity analysis software. The red curve is the fitted combination curve, and the K_d (dissociation equilibrium constant) value is the binding constant of sRNAs and their target mRNAs. The graphs display the data from 4 independent measurements. Green dots, *msrA* versus *DsrO*.

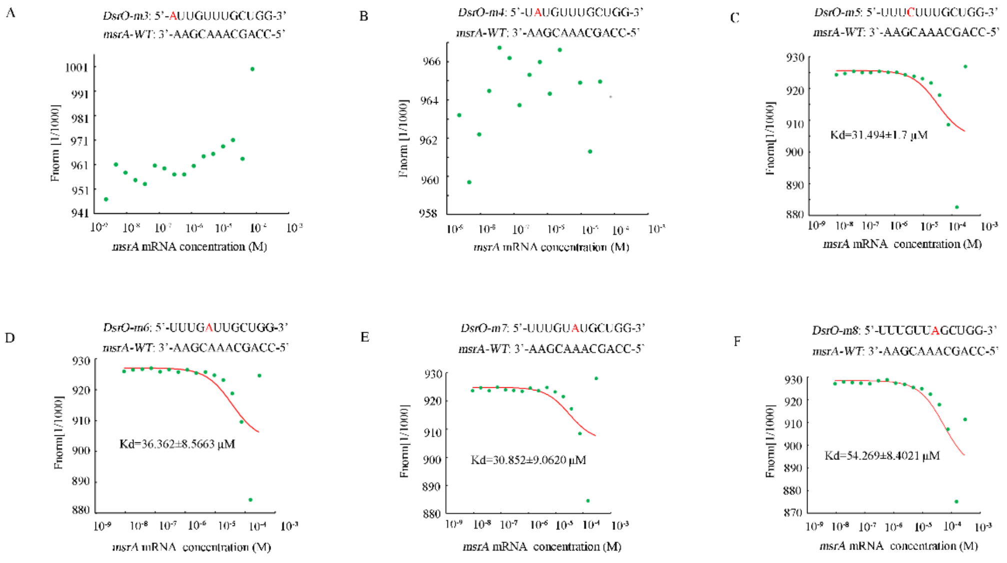

FIG S6 Monitoring binding events between *msrA* and *DsrO.*

A-F. The site-directed mutations of the *DsrO* sequence for determining the interaction ability between *msrA* and *DsrO.* The K_d coefficients were determined for the molecules interaction employing the standard data analysis of MST with affinity analysis software. The red curve was the fitted combination curve, and the K_d (dissociation equilibrium constant) value was the binding constant of sRNAs and their targets. The graphs displayed the data from 4 independent measurements. Green dots, *msrA* versus *DsrO.*

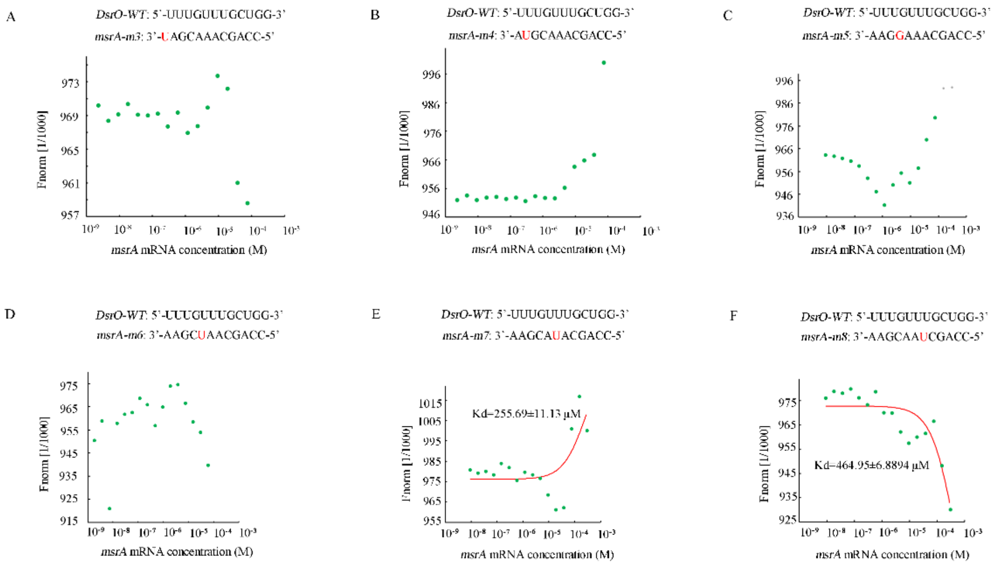

FIG S7 Monitoring binding events between *msrA* and *DsrO.*

A-F. The site-directed mutations of the *msrA* sequence for determining the interaction ability between *msrA* and *DsrO*. The K_d coefficients were determined for the molecules' interaction employing the standard data analysis of MST with affinity analysis software. The red curve was the fitted combination curve, and the K_d (dissociation equilibrium constant) value was the binding constant of sRNAs and their targets. The graphs displayed the data from 4 independent measurements. Green dots, *msrA* versus *DsrO*.
